# Calpain-2 expression is associated with response to platinum based chemotherapy, progression-free and overall survival in ovarian cancer

**DOI:** 10.1111/j.1582-4934.2012.01559.x

**Published:** 2012-09-26

**Authors:** Sarah J Storr, Sabreena Safuan, Caroline M Woolston, Tarek Abdel-Fatah, Suha Deen, Stephen Y Chan, Stewart G Martin

**Affiliations:** a Academic Oncology University of Nottingham School of Molecular Medical Sciences, Nottingham University Hospitals NHS TrustNottingham, UK; b Clinical Oncology, Nottingham University Hospitals NHS TrustNottingham, UK; c Department of Histopathology, Nottingham University Hospitals NHS TrustNottingham, UK

**Keywords:** calpain, ovarian cancer, platinum chemotherapy, calpastatin

## Abstract

Ovarian cancer is routinely treated with surgery and platinum-based chemotherapy. Resistance is a major obstacle in the efficacy of this chemotherapy regimen and the ability to identify those patients at risk of developing resistance is of considerable clinical importance. The expression of calpain-1, calpain-2 and calpastatin were determined using standard immunohistochemistry on a tissue microarray of 154 primary ovarian carcinomas from patients subsequently treated with platinum-based adjuvant chemotherapy. High levels of calpain-2 expression was significantly associated with platinum resistant tumours (*P* = 0.031). Furthermore, high expression of calpain-2 was significantly associated with progression-free (*P* = 0.049) and overall survival (*P* = 0.006) in this cohort. The association between calpain-2 expression and overall survival remained significant in multivariate analysis accounting for tumour grade, stage, optimal debulking and platinum sensitivity (hazard ratio = 2.174; 95% confidence interval = 1.144–4.130; *P* = 0.018). The results suggest that determining calpain-2 expression in ovarian carcinomas may allow prognostic stratification of patients treated with surgery and platinum-based chemotherapy. The findings of this study warrant validation in a larger clinical cohort.

## Introduction

Ovarian cancer is the eighth most common cancer in females, and the seventh leading cause of cancer mortality in women worldwide [1]. Treatment for all stages of ovarian cancer principally consists of surgery and platinum-based chemotherapy. Despite initial sensitivity to platinum-based agents some tumours become resistant, or are already intrinsically resistant to the treatment. Platinum resistance is a major obstacle in the efficacy of platinum-based chemotherapy regimens, and biomarkers of platinum-based chemotherapy response are therefore of substantial clinical interest. There are a number of studies that have investigated biomarkers that have the potential to stratify ovarian cancer patients based on their response to platinum-based chemotherapy. Tissue expression of vascular endothelial growth factor-A is significantly higher in patients who develop chemotherapy-resistant disease and have adverse overall survival [2] and high nuclear and low cytoplasmic expression of thioredoxin and low expression of metallothionein has been shown to be associated with increased progression-free and overall survival in ovarian cancer patients treated with platinum-based chemotherapy; but is not associated with platinum resistance [3]. Platinum-based agents are believed to instigate cell death following induction of DNA adducts, and there is accumulating *in vitro* evidence that platinum-induced apoptosis can be mediated, in part, by calpains [4–6].

The calpain system is an important group of proteases, with micro (μ)-calpain and milli (m)-calpain being the most widely studied family members [7]. These neutral cysteine proteases are responsible for the controlled proteolysis of a number of cellular substrates important in cellular migration, survival and apoptosis. Both μ-calpain and m-calpain are composed of a large 80-kDa catalytic subunit, calpain-1 and calpain-2, respectively, and have a common small 28-kDa regulatory subunit. The enzymes require calcium for their activation, and μ-calpain and m-calpain are named for the *in vitro* calcium concentration required for their activity [7]. There are several mechanisms by which the calcium concentration required for activation can be lowered including protein autolysis and interaction with membrane phospholipids [8, 9]. Furthermore, m-calpain can be phosphorylated by ERK and protein kinase Cι, which has been shown to alter cell adhesion and migration [10, 11]. Calpastatin is the endogenous inhibitor of μ-calpain and m-calpain and can reversibly inhibit up to four calpain heterodimers, with inhibitory action requiring calcium-induced structural changes to calpain [12–14]. Recent evidence has indicated the importance of calpain in cancer progression, in particular during cellular migration, and has highlighted a potential role of the calpain system in altering the response to certain cancer therapies [15]. *In vitro* evidence in ovarian cancer cell lines suggests that calcium release following cisplatin treatment increases calpain activity which can cleave p73 [5]. Importantly, Al-Bahlani *et al*. ([5]), showed that cells with reduced calpain activity, or increased p73 were less sensitive to cisplatin treatment [5].

Due to the importance of calpain activity in tumour progression, its role in other tumour types, and *in vitro* evidence suggesting a role in the response of ovarian cancer cell lines to cisplatin the aims of the current study were to investigate the expression levels of calpastatin, calpain-1 (μ-calpain) and calpain-2 (m-calpain) in tumours from ovarian cancer patients treated with platinum-based adjuvant chemotherapy. Furthermore, it aims to determine the importance of calpain expression in terms of associations with clinicopathological variables and the clinical outcome of the patients. Importantly a measure of response to platinum-based chemotherapy was available for all the patients in the form of platinum resistance or sensitivity determined clinically, defined as those patients that progressed during first line chemotherapy.

## Materials and methods

### Clinical cohort

The clinical cohort comprised of 154 patients with primary ovarian cancer treated with carboplatin-based adjuvant chemotherapy at Nottingham University Hospitals between 2000 and 2007. Forty-two (65/154) of patients received carboplatinum alone, whereas the remaining patients received combined platinum regimens, including 78 of 89 (88%) with paclitaxel and the remaining 11 of 89 (12%) patients receiving alternate combinations (carboplatin in combination with gemcitabine, topotecan or liposomal doxorubicin) dependent upon their inclusion in concurrent clinical trials (ICON trial). Ethical approval was obtained from the Derbyshire ethics committee (06/Q2401/153). Clinicopathological data such as tumour grade and stage were retrospectively recorded for the cohort and are shown in Table 1. The mean age of the cohort was 59.7 years ranging in years from 33 to 87. Progression-free survival was calculated from the date of the initial surgery to disease progression or from the date of the initial surgery to the last date known to be progression-free for those censored. Overall survival time was calculated from the date of the initial surgery to death or from date of the initial surgery to last date known to be alive for those censored. The study was retrospectively analysed up to 30 April 2009 and at this point 51% (79/154) of patients had died and 57% (85/150) of patients had progressive disease. The median follow-up for this study was 48 months. Platinum resistant cases were defined as those patients that progressed with first-line platinum-based chemotherapy during treatment, or within 6 months of treatment. Thirty-two% (50/154) of patients had platinum-resistant disease. This study is reported in concordance with REMARK criteria [16]

**Table 1:** Associations between calpastatin, calpain-1 and calpain-2 protein expression and various clinicopathological variables. The frequency of observed clinicopathological variables is noted next to the variable subgroup. The *P*-values are resultant from Pearson chi-square test of association (χ^2^) or Fisher's exact test in a 2 × 2 table if a cell count was less than 5 (indicated by *). Significant *P*-values are indicated by bold font

Variable	Calpastatin			Calpain-1			Calpain-2		
		
	Low	High	*P*-value	Low	High	*P*-value	Low	High	*P*-value
Pathology									
Serous (*n* = 91)	11	56	**<0.001**	5	62	**0.003**	13	55	**<0.001**
Mucinous (*n* = 11)	5	3		0	8		4	4	
Endometrioid (*n* = 29)	9	15		7	18		9	15	
Clear cell (*n* = 20)	11	6		7	10		14	3	
Others (*n* = 3)	2	0		0	1		0	1	
Grade									
1 (*n* = 19)	5	10	0.556	2	13	0.925	4	10	0.313
2 (*n* = 22)	3	12		2	12		3	13	
3 (*n* = 113)	30	58		15	74		33	55	
Debulking surgery									
Optimal (*n* = 90)	27	44	0.096	13	57	0.378	27	42	0.154
Suboptimal (*n* = 64)	11	36		6	42		13	36	
Stage									
I (*n* = 42)	16	19	0.105	8	27	0.637	17	17	0.103
II (*n* = 20)	3	11		2	12		4	10	
III (*n* = 71)	17	37		7	46		16	38	
IV (*n* = 21)	2	13		2	14		3	13	
Chemotherapy									
Carboplatin monotherapy (*n* = 60)	20	30	0.103	10	41	0.405	19	30	0.315
Carboplatin combination (*n* = 88)	17	49		9	56		20	47	
Platinum sensitivity									
Sensitive (*n* = 104)	27	54	0.698	15	66	0.419*	32	47	**0.031**
Resistant (*n* = 50)	11	26		4	33		8	31	
Progression-free status									
progression-free (*n* = 65)	22	32	0.064	10	45	0.646	23	29	0.053
progressive disease (*n* = 85)	15	46		9	51		17	46	
Survival status									
Living (*n* = 75)	19	43	0.703	9	54	0.566	27	34	**0.014**
Deceased (*n* = 79)	19	37		10	45		13	44	

### TMA construction and immunohistochemistry

The ovarian cancer tissue microarrays (TMAs) were prepared as described previously, with two tumour tissue cores assessed per patient [3]. The tumour cores that were assessed in this study was not selected upon tumour location, but that they were representative of the tumour. Four micrometre sections of the TMA were mounted on poly-l-lysine coated slides. Immunohistochemistry was performed using a similar method to that previously described for breast cancer using the primary antibodies; mouse anti-calpastatin, mouse anti-calpain-1 and rabbit anti-calpain-2 [all Chemicon (Millipore, MA, USA) clones PI-11, P-6 and rabbit polyclonal AB1625, respectively, with specificity confirmed by Western blotting] [17, 18]. Briefly slides were deparaffinized in histolene followed by rehydration in a series of ethanol baths (100%, 90%, 70%, 50% and 30% in water). Antigen retrieval was performed in pre-heated 0.01 mol/l sodium citrate buffer (pH6) in a microwave at 400 W for 10 min. Endogenous peroxidase activity was blocked over 10 min. in 0.01% hydrogen peroxide in methanol. Staining was achieved using the Vectastain Elite ABC kit (universal), containing blocking serum, biotinylated secondary antibody and ABC reagent (Vector Laboratories, Peterborough, UK). Primary antibodies were diluted in blocking serum (calpastatin 1:15,000; calpain-1 1:2500; calpain-2 1:2500) and applied to the tissue for 1 hr at room temperature. Immunohistochemical reactions were developed with 3,3′ diaminobenzidine as the chromogenic peroxidase substrate (Dako, Glostrup, Denmark). Sections were then counterstained with Gills formula Haematoxylin (Vector Laboratories), dehydrated and fixed in histolene prior to mounting with DPX. Breast tumour composite sections which comprised of six stage 1 breast tumours of grade 1–3 were included as positive and negative controls with each run, with the negative control having primary antibody substituted for PBS.

Assessment of staining was conducted after scanning of the slides with a Nanozoomer Digital Pathology Scanner (Hamamatsu Photonics, Hamamatsu City, Japan) at 20× magnification. Calpastatin, calpain-1 and calpain-2 expression in tumour cells was manually assessed using an immunohistochemical H-score as described previously [17]. Briefly, staining intensity of tumour cells was assessed as; none (0), weak (1), medium (2) and strong (3) over the percentage area of each staining intensity. H-scores were calculated by multiplying the percentage area by the intensity grade (H-score range 0–300). A minimum of 30% of tissue cores were examined by a second independent assessor, with the majority of patients having one tissue core assessed by both scorers. Both assessors were blinded to each other's scores and clinicopathologic criteria and there was good concordance between both scorers (single measure intra-class correlations greater than 0.75). Each case had two cores assessed and an average H-score calculated. The continuous data was dichotomized for analysis using X-tile [19].

### Statistical analysis

The relationship between categorized protein expression and clinicopathologic factors was analysed using Pearson chi-square test of association (χ^2^) or Fisher's exact test in a 2 × 2 table if a cell count was less than 5. Overall disease specific survival curves were plotted according to the Kaplan–Meier method and significance determined using the log-rank test. Multivariate survival analysis was performed by Cox Proportional hazards analysis. All differences were deemed significant at the level of *P* < 0.05. Statistical analysis was performed using spss 17.0 software (IBM corporation, NY, USA).

## Results

### Immunohistochemistry

**Fig 1:**
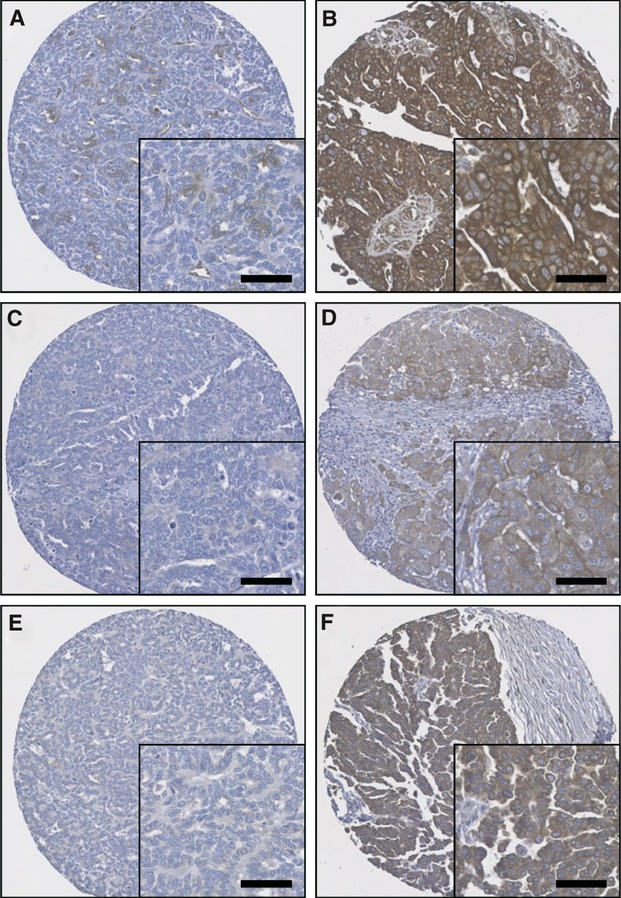
Representative photomicrographs of protein expression levels. (A) low calpastatin expression (H-score 60); (B) high calpastatin expression (H-score 280); (C) low calpain-1 expression (H-score 10); (D) high calpain-1 expression (H-score 200); (E) low calpain-2 expression (H-score 20); (F) high calpain-2 expression (H-score 165). Photomicrographs are at 10× magnification with 20× magnification inset box where scale bar shows 50 μm.

Tissue expression of calpastatin, calpain-1 and calpain-2 was assessed in a series of 154 ovarian cancer patients treated with platinum-based chemotherapy. All three proteins were mainly located in the cytoplasm, with some granularity and heterogeneity between adjacent tumour cells, varying from weak to intense staining. Nuclear staining was observed quite frequently for calpastatin. Some stromal cell staining was observed, however, this was not scored as part of this study. Calpastatin had a mean H-score of 184 and a standard deviation of ±67; calpain-1 had a mean H-score of 114 ± 74 and calpain-2 had a mean H-score of 159 ± 47. Representative staining is shown in photomicrographs in Fig. 1. Two cores were assessed for each patient, with an average H-score being used for analysis. The correlation between the two scores for each patient was evaluated using Spearman's rank correlation coefficient. A significant correlation between the two scores for expression of calpain-1, calpain-2 and calpastatin was observed.

### Relationship with clinicopathological variables

Calpastatin, calpain-1 and calpain-2 H-scores were dichotomized using X-tile software into low and high immunoreactivity and were tested for associations with clinicopathologic criteria. X-tile generated cut points were as follows: calpastatin had a H-score cut point of 165 with 80 cases having a high score; calpain-1 had a H-score cut point of 25 with 99 cases having a high score; calpain-2 had a H-score cut point of 162 with 78 cases having a high score. A small number of TMA cores were not assessed due to missing cores or insufficient representative tumour. Associations between protein expression and clinicopathological criteria are shown in Table 1. Ovarian tumours of the serous type were associated with high expression of calpastatin (χ^2^ = 23.755, d.f. = 4, *P* < 0.001), high calpain-1 (χ^2^=15.961, d.f. = 4, *P* = 0.003) and high calpain-2 (χ^2^ = 26.02, d.f. = 4, *P* < 0.001). High calpain-2 expression was associated with platinum resistant tumours (χ^2^ = 4.658, d.f. = 1, *P* = 0.031) and with death of the patient (χ^2^ = 6.053, d.f. = 1, *P* = 0.014).

### Relationship with progression-free survival

**Fig 2:**
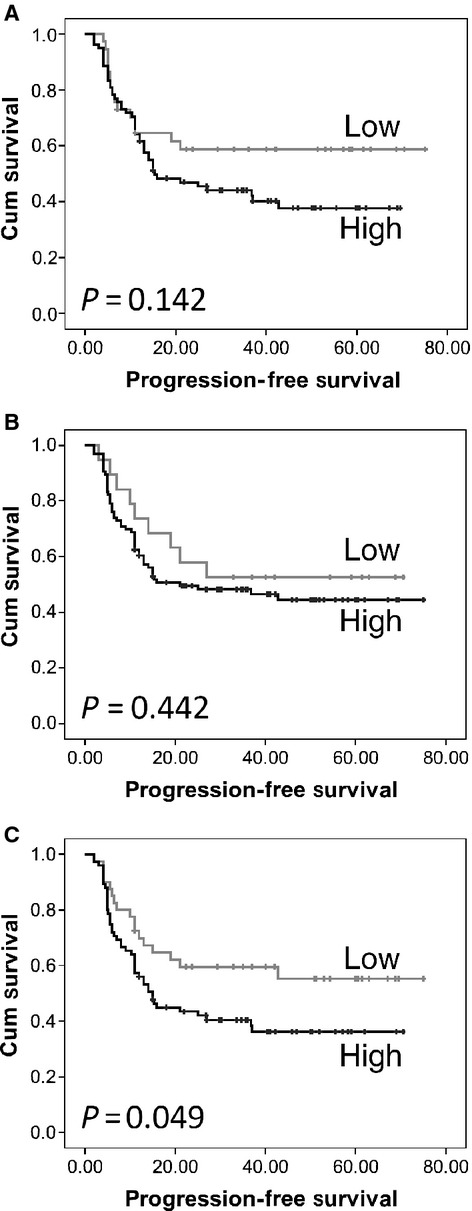
Kaplan–Meier analysis of progression-free survival showing the impact of calpastatin (A), calpain-1 (B) and calpain-2 (C) expression in cohort of 154 patients with significance determined using the log rank test. (A) low expression group has 37 observations and 15 events and high expression group has 78 observations and 46 events; (B) low expression group has 19 observations and 9 events and high expression group has 96 observations and 51 events; (C) low expression group has 40 observations and 17 events and high expression group has 75 observations and 46 events; where observations are the number of observations per group and events are those patients with progressive disease.

At the end of the follow-up period 85 patients had developed progressive-disease, with a median progression-free survival of 14 months. The expression of calpastatin and calpain-1 was not associated with overall or progression-free survival (Fig. 2A and B). However, high expression of calpain-2 was significantly associated with progression-free survival (*P* = 0.049) (Fig. 2C). In multivariate analysis, including grade, stage, optimal debulking and platinum sensitivity, calpain-2 expression was not an independent marker of progression-free survival (Table 2A). Tumour grade, stage, optimal debulking and platinum sensitivity were included in multivariate analysis following assessment using the Kaplan–Meier method and significance determined using the log-rank test with individual values of *P* = 0.004, *P <* 0.001, *P* < 0.001, and *P* < 0.001 respectively.

**Table 2:** Cox proportional hazards analysis for progression-free (A) and overall-survival (B) for calpain-2 expression. Exp (B) is used to denote hazard ratio, and 95% CI is used to denote 95% confidence interval

	*P*-value	Exp (B)	95% CI for Exp (B)	

			Upper	Lower
(A) Progression-free survival				
Calpain-2 expression	0.880	0.955	0.524	1.739
Grade	0.254	1.320	0.820	2.124
Stage	0.001	1.891	1.321	2.706
Optimal debulking	0.861	1.057	0.570	1.961
Platinum sensitivity	0.000	9.025	4.696	17.345
(B) Overall survival				
Calpain-2 expression	0.018	2.174	1.144	4.130
Grade	0.060	1.676	0.978	2.873
Stage	0.205	1.265	0.879	1.819
Optimal debulking	0.476	1.258	0.669	2.368
Platinum sensitivity	0.000	4.938	2.462	9.904

### Relationship with overall survival

**Fig 3:**
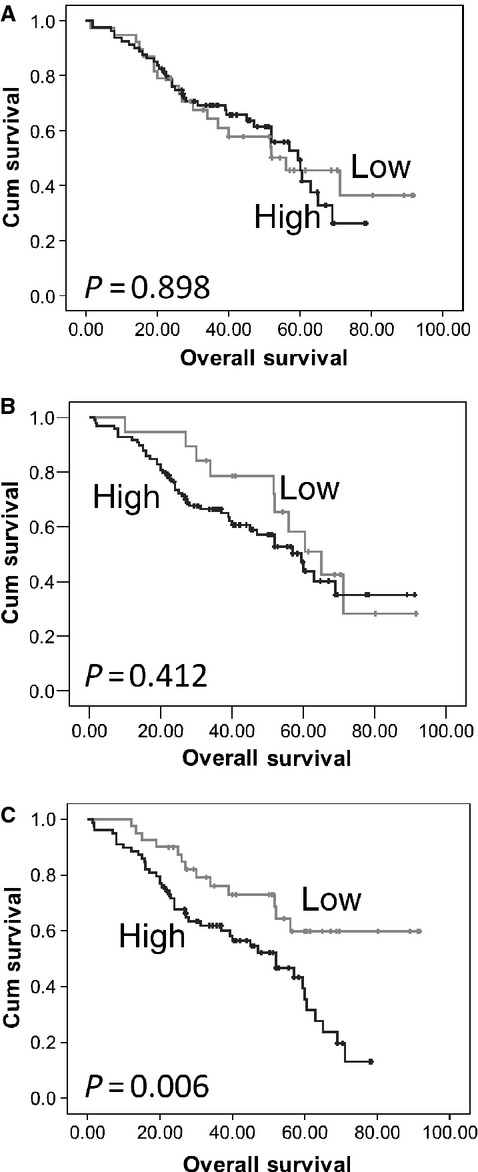
Kaplan–Meier analysis of overall survival showing the impact of calpastatin (A), calpain-1 (B) and calpain-2 (C) expression in cohort of 154 patients with significance determined using the log rank test. (A) low expression group has 38 observations and 19 events and high expression group has 80 observations and 37 events; (B) low expression group has 19 observations and 10 events and high expression group has 99 observations and 45 events; (C) low expression group has 40 observations and 13 events and high expression group has 78 observations and 44 events; where observations are the number of observations per group and events are those patients who died.

At the end of the follow-up period 79 patients had died, with a median survival of 35 months. The expression of calpastatin and calpain-1 was not associated with overall survival (Fig. 3A and B). High expression of calpain-2 was significantly associated with overall survival (*P* = 0.006) (Fig. 3C). In multivariate analysis, including grade, stage, optimal debulking and platinum sensitivity, calpain-2 expression was independently significant for overall survival (hazard ratio = 2.174; 95% confidence interval = 1.144–4.130; *P* = 0.018) (Table 2B). Tumour grade, stage, optimal debulking and platinum sensitivity were included in multivariate analysis following assessment using the Kaplan–Meier method and significance determined using the log-rank test with individual values of *P* = 0.008, *P* < 0.001, *P* < 0.001 and *P* < 0.001 respectively.

## Discussion

The expression levels of calpastatin, calpain-1 and calpain-2 were determined in a cohort of 154 ovarian cancer patients treated with platinum-based adjuvant chemotherapy. No prior studies have investigated the expression of calpastatin, calpain-1 and calpain-2 in ovarian cancer, or have tested their association with response to platinum-based chemotherapy in tissue samples. The expression levels of these proteins have previously been investigated in breast cancer, with results indicating that expression of calpain-1 is associated with relapse-free survival in HER2 positive breast cancer patients treated with trastuzumab following adjuvant chemotherapy [17].

The calpain system, including the proteolytic enzymes μ-calpain and m-calpain and their endogenous inhibitor calpastatin, has been implicated in cancer progression and response to therapy in a limited number of studies and tumour types [15]. Recent *in vitro* evidence has suggested that calpain activity following cisplatin treatment influences the apoptotic process by reducing p73 which acts as a pro-apoptotic factor [5]. In the current study, high calpain-2 expression is of clear adverse importance in ovarian cancer patients. Although the *in vitro* evidence suggests calpain activity is important in cisplatin-induced apoptosis, translational evidence suggests high calpain-2 expression is important in platinum-based therapy resistance. There are a large number of differences between studies; the *in vitro* study investigated calpain modulation in a range of ovarian cancer cell lines at the time of treatment with cisplatin; whereas the current study documents the expression level of calpain-2 in tumours prior to treatment. High expression of calpain-2 in the surgically excised tumour may indicate that the tumour, and by implication any tumour cells remaining after surgery, has evolved mechanisms of surviving high calpain-2 expression. In this manner, high calpain-2 expression may be beneficial to cell survival by making calpain-dependent apoptosis ineffective in the face of systemic platinum-based therapy; alternatively high calpain-2 expression in the primary tumour may suggest that the tumour has a higher capacity for migration, and that these tumours may have a more aggressive phenotype. The role of calpain in altering cell migration is well established. Evidence has shown that calpain can cleave a number of substrates involved in promoting cellular motility, including focal adhesion kinase (FAK) and talin but also many other substrates such as paxillin, fodrin and ezrin [20–24]. It should be noted that this study investigated the relative protein expression levels of calpain-1 and calpain-2, and not their activity within the tissues. As part of future studies it would be interesting to assess calpain activity within ovarian carcinoma tissue using antibodies against calpain-specific digestion products, however, such antibodies require validation and characterization for use in human cancer [25, 26]. Interestingly the cut-point for calpain-1 was lower than that of calpain-2; it remains unclear as to why this would occur although the lower calcium concentration required for calpain-1 activation may mean that lower expression levels of the protein are required.

Although there was a significant association between high calpain-2 expression and platinum sensitivity, high calpain-2 expression was only marginally associated with progression-free survival (*P* = 0.049), but clearly associated with overall survival (*P* = 0.006) which remained significant in multivariate analysis. This finding was perhaps unexpected as although platinum sensitivity is significantly associated with overall survival (*P* = 3.64 × 10^−18^) its link with progression-free survival is even stronger (*P* = 3.77 × 10^−37^). The association between high calpain-2 expression and overall survival is independent of platinum sensitivity which suggests that any effect calpain-2 expression exerts upon clinical endpoint is not solely due to modulation of the cellular response to platinum-based chemotherapy. Advanced mucinous ovarian carcinoma has been shown to have a poorer response to platinum-based chemotherapy [27, 28]. In this study, we do observe a difference between histological type and platinum resistance (χ^2^ = 13.724, d.f. = 4, *P* = 0.008), however, there are insufficient cases of alternate histological subtype to perform subset analysis of calpain expression and clinical outcome in these groups.

In summary, high expression of calpain-2 is significantly associated with resistance to platinum-based adjuvant chemotherapy. Furthermore, high calpain-2 expression is significantly associated with both poor progression-free and overall survival. The association between high calpain-2 expression and overall survival remains significant in multivariate analysis. Determining calpain-2 expression in ovarian cancer patients prior to platinum-based chemotherapy may identify those patients whose tumours are at high risk of developing resistance to the chemotherapy regimen and identification of such individuals will allow more rigorous follow-up or alternate treatment regimens to be considered. The findings of this study warrant validation in a larger clinical cohort.
